# Promoting physical activity in worksite settings: results of a German pilot study of the online intervention Healingo fit

**DOI:** 10.1186/s12889-017-4697-6

**Published:** 2017-09-08

**Authors:** Kevin Dadaczynski, Stephan Schiemann, Olaf Backhaus

**Affiliations:** 1 Centre for Applied Health Science, Leuphana University Lueneburg, Wilschenbrucher Weg 84a, 21335 Lueneburg, Germany; 2 Federal Center for Health Education, Cologne, Germany; 3 Institute of Sport Science, Leuphana University Lueneburg, Lueneburg, Germany; 4 Department of Social Work and Health, University of Applied Science Kiel, Kiel, Germany

**Keywords:** Physical activity, Worksite health promotion, Online intervention, Pedometer, Gamification, RCT

## Abstract

**Background:**

Worldwide, one third of the adult population is insufficiently physically active. This fact has led to a strong demand for public health initiatives. Given the mixed evidence on the effectiveness of worksite interventions promoting physical activity (PA), a pedometer-based and gamified intervention, Healingo Fit, was developed and evaluated over a period of six weeks.

**Methods:**

The effectiveness of Healingo Fit was evaluated as part of a randomized controlled trial (RCT) with two measurement points involving employees of an automobile manufacturer. Direct health promotion outcomes were assessed using self-developed items on PA knowledge, the HAPA brief scales and the exercise self-efficacy scale. IPAQ short version was used to assess different forms of PA behavior. Intervention effects were identified using a two-way analysis of variance (ANOVA) with repeated measurements.

**Results:**

A total of 144 participants took part in the study (intervention group = 80, control group = 64). The results of the ANOVA show significant interaction effects (group x time) for health promotion outcomes (knowledge, intention, and self-efficacy), with medium to high effect sizes. In the health behavior related outcomes, there were significant improvements, with large effect sizes for low levels of PA, but not for moderate and high PA. Walking time increased by 125 min/week in the intervention group, corresponding to a percentage increase of 30% compared to baseline.

**Conclusions:**

Pedometer-based interventions using gamification elements can have positive effects not only on health promotion parameters but can also lead to an increase in PA behavior. The online format of Healingo Fit is suitable for reaching large numbers of people and achieving population effects.

**Trial registration:**

German Clinical Trials Register (DRKS): DRKS00006105, date of registration: 2017–03-24.

**Electronic supplementary material:**

The online version of this article (10.1186/s12889-017-4697-6) contains supplementary material, which is available to authorized users.

## Background

There is sufficient evidence that physical activity (PA) plays an important role in preventing modern civilization diseases. Numerous studies demonstrate significant associations between PA and cardiovascular diseases including its risk factors (e.g. high blood pressure, obesity, diabetes) [1], various cancerous diseases [2], musculoskeletal disorders [3], mental disorders and conditions [4], and an increase in mortality probability [5]. According to the World Health Organization (WHO), PA is the fourth leading mortality risk factor and is responsible for an estimated 3.2 million deaths per year worldwide [6].

The high public health relevance of PA is facing alarming findings regarding the PA behavior of adults. Worldwide, epidemiological findings from 122 countries show, that almost one third of the adult population (15 years or older) are physically inactive with lowest rates found in Southeast Asia and highest in the USA and the Mediterranean countries [7]. According to a representative German study only 44% of males and 35% of females over 18 years are engaged in activities for 2.5 h or more that are vigorous enough to cause them to be out of breath or to sweat [7]. In general, PA levels are higher for females, older people, and for adults from highly industrialized countries [7, 8].

Given the importance of PA for health coupled with high rates of physical inactivity, it has become increasingly urgent to develop effective measures to promote PA. The workplace has been identified as an appropriate setting for health promotion as individual level measures which aim to support PA have the potential to reach a substantial proportion of adults who are employed [9]. Studies evaluating their effectiveness are however contradictory. Recent reviews show that 40% to 45% of the included intervention studies do not show positive effects on PA [10, 11]. However, even if there is uncertainty regarding the effectiveness of different intervention types, their methods, and implementation, the use of pedometers as an intervention tool was shown to be promising. In seven of the 20 studies reviewed by Quyen et al. [10], a pedometer was used and their evaluation showed a significant increase in or a reduced decrease in the number of steps taken. Furthermore, in a systematic review of 26 studies the use of pedometers showed an average increase in PA of 26.9% in a comparison to baseline measurement and a control group [12].

Alongside the use of pedometers, a further promising innovation in exercise-related health promotion and disease prevention is the employment of gaming approaches. While screen time (e.g. digital games) has usually been considered a risk factor for health [13, 14], new approaches such as “serious games” and “exergames” (exercise games) have been explicitly developed and implemented with the objective of promoting PA [15]. Recent literature reviews conclude that such gaming applications lead to increased energy expenditure and greater levels of low and moderate PA [15, 16]. Although there are currently a number of applications for therapeutic use, there is also a lack of methodologically sound worksite health promotion interventions.

Against this background, Healingo Fit (**Heal**th **In**tegrated **G**aming **O**nline) was developed as a tracking-based intervention for the promotion of PA using of a number of game elements. The effectiveness of the intervention was evaluated in a randomized controlled trial (RCT) in a worksite setting.

## Methods

### Study design and sample

An intervention group (IG) and a waiting-list control group (CG) (who did not receive the intervention until the study was ended) were studied in cooperation with a German automobile manufacturer using a RCT with online-based evaluation assessments at the start and after six weeks. The study had a two-factor design, with a group factor (intervention group [IG] versus control group [CG]) and a two-way repeated measures factor (t_1_ = pre-intervention, t_2_ = post-intervention). Participants were eligible to participate if they were 18 years or older, had a permanent contract (no temporary workers) and had no pre-existing health condition. As the intervention is conceived as a universal prevention program there were no further inclusion and exclusion criteria. Participants were recruited by the health service of the cooperating company, e.g. through distribution of flyer, employee TV channel, intranet, and oral presentation of the intervention in employee meetings. Interested employees were admitted and randomly assigned to the intervention or to the control group using a computerized random number generator. This randomization ensured pretest equivalence of all variables. The study protocol was approved by the ethics committee of Leuphana University Lueneburg and registered at the German Clinical Trials Register.

**Fig. 1:**
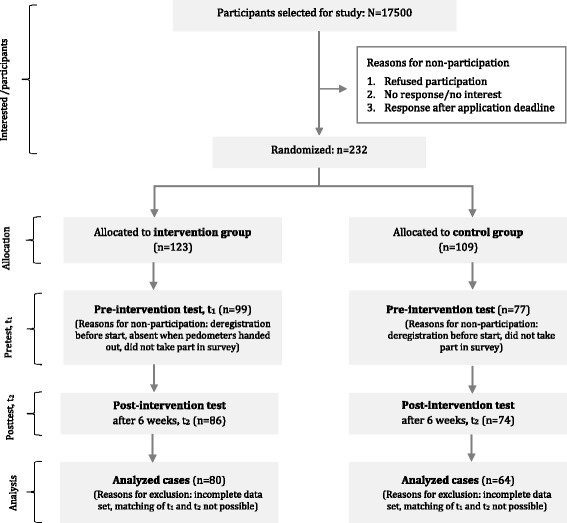
Consort diagram: Participation and allocation in the Healingo Fit intervention program

A total of 232 persons expressed their interest in participating in the study, with 176 ultimately taking part (IG = 99; CG = 77) (Fig. 1). At the beginning of the intervention the participants of the IG were instructed about the intervention according to a standardized procedure. This involved distributing an information brochure about the study, issuing and setting up a pedometer, as well as linking it to the online intervention Healingo Fit.

Participants were invited by email to take part at an online survey at pre and post measurement point. Upon entering the survey site, participants were presented with information regarding the parameters of the study in order to facilitate informed consent. After checking a consent box at the bottom of the page participants were directed to the questionnaire.

A total of 160 persons (IG = 86; CG = 74) took part in the post-intervention survey. As only those cases were analyzed that could be identified as taking part at both measurement points, the final sample comprised 144 persons (IG = 80; CG = 64).

Generally, males (IG = 61.3%, CG = 68.8%), participants with vocational training (IG = 66.3%, CG = 67.2%) as well as full-time employees (IG = 91.3%, CG: 90.6%) were over-represented in the sample. However, a comparison of the socio-demographic data using the chi-square test does not show significant differences between IG and CG (Table 1).

**Table 1:** Sociodemographic data of the study sample (frequency in %)

	Intervention group (*n* = 80)	Control group (*n* = 64)	*p*
Sex			
Male	61.3	68.8	.350 (ns)
Female	38.8	31.3	
Age			
≤ 35 years old	30.0	43.8	.187 (ns)
36 to 45 years old	30.0	28.1	
≥ 46 years old	40.0	28.1	
Educational status			
No/in vocational training	10.0	6.3	.699 (ns)
Vocational training	66.3	67.2	
Higher education	23.8	26.6	
Extent of employment			
Full time	91.3	90.6	.703 (ns)
Part time	8.7	9.4	

*p* = significance (determined using chi-square test), ns = not significant

### The online intervention Healingo fit

**Fig. 2:**
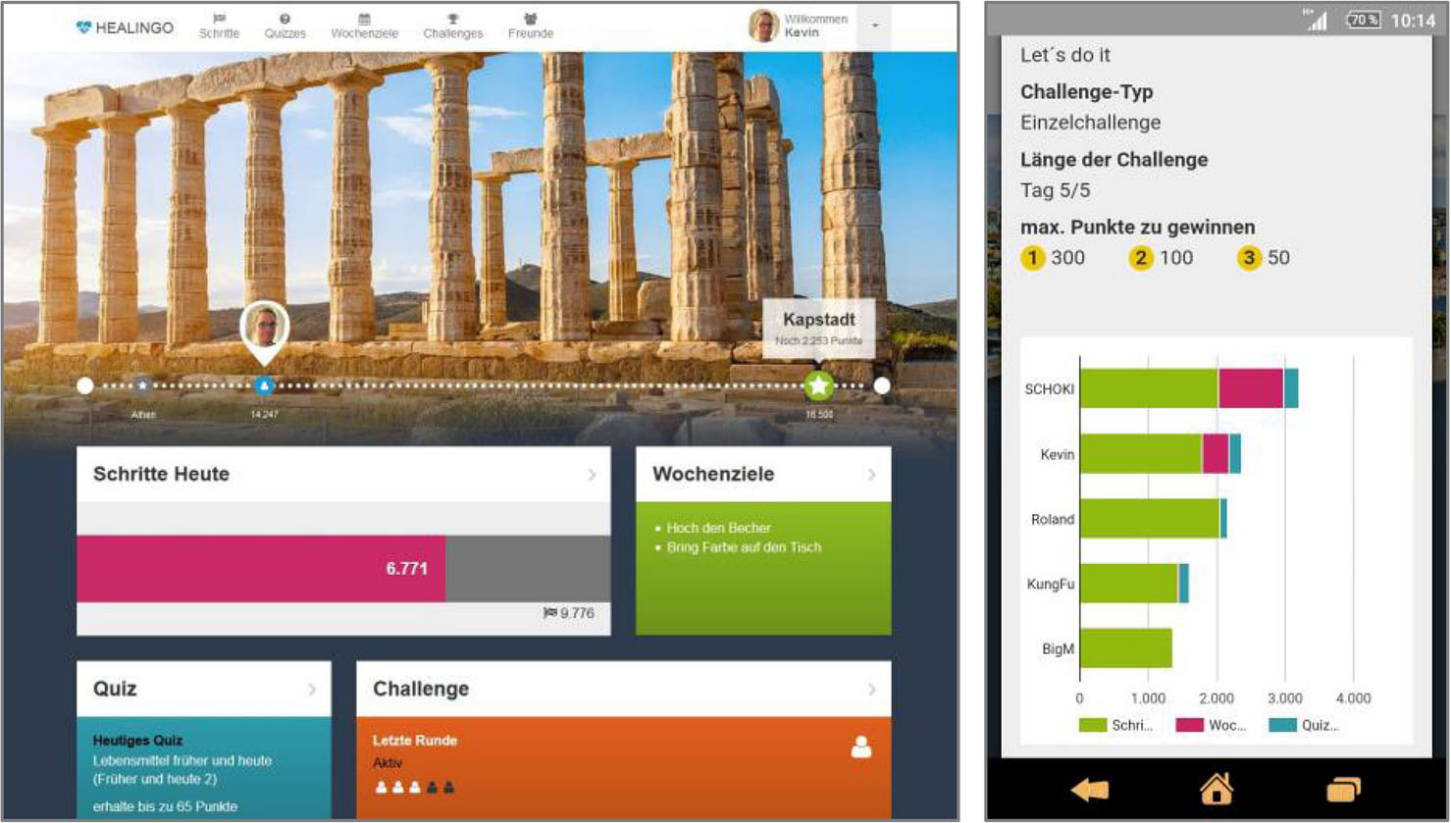
Screenshot of the online intervention Healingo Fit (left: desktop version, right: mobile version)

Healingo Fit is a universal prevention intervention, with the main objective to promote low levels of PA (e.g. walking activities). This was accomplished by using a tracking-based approach measuring PA with a Fitbit® pedometer (Fitbit Zip) implemented in a six-week browser-based online intervention, which could be accessed by desktop and mobile devices. Based on socio-cognitive learning theory [17], the theory of planned behavior [18] and the health action process approach (HAPA) model [19], Healingo Fit used a wide range of behavior change methods that aimed at PA related knowledge (e.g. provision of information), intentions (e.g. goal setting), volitional factors (e.g. action-planning), self-efficacy (e.g. self-monitoring and positive feedback) as well as subjective norms and role models (e.g. social encouragement, social comparisons). For this purpose four modules were developed and integrated into a coherent intervention concept (Fig. 2):

1. **Steps and step goals:** Following international PA recommendations [20], a daily step goal was implemented. In contrast to conventional static targets, individual tailored step goals were calculated each day based on logged data of the pedometer for each participant of the last four days. Participants who complied with or exceed the PA recommendations were instructed to maintain this level.
2. **Quizzes:** A quiz module was implemented to increase knowledge on PA and general health. The quiz module included three daily multiple choice questions about PA but also on related topics such as on healthy diet (e.g.: What is the intensity of a healthy endurance training? Which food contains a lot of vitamin A? Which of the following criteria should be considered when setting goals for one’s own health?). In addition to individual feedback, the participants received detailed information about the areas they were quizzed on. This information could be accessed during the whole intervention period.
3. **Health goals:** Participants could simultaneously choose as many as three goals (from a list of 60 pre-defined goals) and follow them over a period of seven days. In addition to PA, the goals also comprised topics on e.g. healthy eating (e.g. Stair Star: Challenge yourself and use the entire day the stairs instead of the elevator; Fruit Fighter: Eat at least 3 servings of fruit per day.). This module also included a self-evaluation of the participant’s success in reaching his or her goals and the provision of a tip that could be read and evaluated by other participants.
4. **Challenges:** During each intervention week, the participants could join in team or individual challenges against each other. This was based on the participant’s success in implementing modules 1 to 3 (steps, quizzes, health goals), which was documented and visualized over a period of five days.

Each module was connected to the others by means of a thematic game framework. Participants were put in the role of a virtual traveler whose task is to visit 16 cities (representing different levels) over the entire intervention period. Following the gamification approach [21] classic game design elements were implemented to increase participation rates. These included points (for modules 1 to 4), badges as achievement awards, the implementation of a level structure (cities) as well as rankings (leader boards) and different ways of visualizing the progress of the users (e.g. a map). During the intervention period, participants received a daily email with information about the current step goal as well as the results of the previous day (e.g. quizzes solved or points obtained).

### Instruments

In order to detect intervention effects, we used Nutbeam’s outcome model as an evaluation framework which assumes that interventions gradually unfold their effects [22]. Health promotion outcomes are defined as results that can be detected immediately after the intervention. PA related knowledge, intentions, and self-efficacy served as those direct health promotion outcomes. Four items were self-developed to assess the degree of knowledge of PA recommendations. PA-related intentions were assessed using a sub-scale of the HAPA Brief Scales [23] containing three items about the intention to be physically active in the future. Moreover, the exercise self-efficacy scale [24] including four items was used to assess participant intention to put planned PA into practice even when faced with obstacles. As the scale on intention and self-efficacy was only available in English, it was translated and then back-translated by a native speaker to assure content validity. The internal consistency of the three scales were all acceptable to good (.72 < α < .92).

Intermediate health outcomes are defined as determinants of health and represent a second level of Nutbeam’s outcome model which is influenced by direct health promotion outcomes. PA behavior served as an intermediate outcome parameter which was assessed using the German short version of the International Physical Activity Questionnaire (IPAQ-SF, [25]). This questionnaire was developed to gather data about weekly PA of adults between the ages of 15 and 69. Over seven items, participants were asked to self-assess how many days out of the last seven days they did vigorous, moderate or walking activities and how many hours and minutes they spent on each particular PA level on any given day. The test-retest reliability was good (.86 < rtt < .88). An overview of the instruments used in this study is given in Table 2.

**Table 2:** Overview of scales and items

Scale	N_items_	Example	Scale range
PA-related knowledge	4	I am familiar with current recommendations about physical activity.	1–4
PA-related intention (23)	3	I intend to practice physical exercise on a regular basis.	1–4
PA-related self-efficacy (24)	5	I can manage to carry out my exercise intentions … even when I am busy.	1–4
PA-related behavior (25)	7	During the last 7 days, on how many days did you do moderate physical activities like carrying light loads, bicycling at a regular pace, or doubles tennis?	*

*Notes*: n_items_ = number of items, *no uniform response format

### Statistical analyses

In order to investigate the impact of Healingo Fit on health promotion and intermediate outcome parameters, a two-way analysis of variance (ANOVA) with repeated measures was used with the factors “group” and “time”. All necessary prerequisites, e.g. interval-scaled variables and normal distribution of the data, were met. As six outcome parameters were calculated, the alpha level was corrected using the Bonferroni correction (*p* = .05/6 ≈ .008). Partial eta squared (*η*_p_^2^) was used in addition as an effect size measure. The effect size was interpreted using the following conventions: small effect (*η*_p_^2^ ≥ 0.01), medium effect (*η*_p_^2^ ≥ 0.06), large effect (*η*_p_^2^ ≥ 0.14) [26]. All statistical analysis were conducted using IBM© SPSS© statistics software.

## Results

Descriptive statistics for the outcome variables are shown in Table 3 separately for the factors group and measurement point. The mean differences for IG reveal substantial improvements for the health promotion as well as the intermediate outcomes.

**Table 3:** Mean (*M*), standard deviation (*SD*) for PA-related knowledge, attitude and behavior

Outcome		Intervention Group (IG) (*n* = 80)			Control Group (CG) (*n* = 64)		
		*t*_*1*_	*t*_*2*_	*t*_*1*_ *- t*_*2*_	*t*_*1*_	*t*_*2*_	*t*_*1*_ *- t*_*2*_
PA-related knowledge	*M*	1.96	3.28	−1.32	2.09	2.06	0.03
	*SD*	.603	.541		.771	.678	
PA-related intention	*M*	3.29	3.63	−0.34	3.21	3.18	0.03
	*SD*	.642	.523		.473	.500	
PA-related self-efficacy	*M*	2.65	3.02	−0.37	2.70	2.65	0.05
	*SD*	.731	.664		.806	.657	
Vigorous physical activity (min./week)	*M*	217.10	222.32	−5.22	217.06	219.31	−2.25
	*SD*	104.37	112.23		97.60	89.01	
Moderate physical activity (min./week)	*M*	290.75	310.92	−20.17	294.57	291.81	2.76
	*SD*	135.64	129.40		129.54	114.01	
Minutes walked (min./week)	*M*	401.55	526.48	−124.93	424.07	442.87	−18.8
	*SD*	208.03	240.56		203.44	200.36	

t_1_ = before intervention start, t_2_ = immediately after intervention (t_1_ plus 6 weeks), t_1_-t_2_ = average distance between measurement points

As hypothesized, an increase in PA related knowledge in the IG between t_1_ (*M* = 1.96, *SD* = .603) and t_2_ (*M* = 3.28, *SD* = .541) could be found. By contrast, the mean values in the CG between t_1_ (*M* = 2.09, *SD* = .771) and t_2_ (*M* = 2.06, *SD* = .678) remained stable. The interaction effect (group x time) is significant with a large effect size (*F*(1, 142) = 229.52, *p* < .008, *η*p^2^ = .618) (Table 4 & Additional file 1: Fig. S1). At the level of single items, the greatest increase of knowledge can be found with regard to the number of steps to be taken for an active lifestyle (t_1_-t_2_ = −1.74) followed by knowledge about the number of steps associated with specific activities (e.g. go for a 30 min’ walk, t_1_-t_2_ = −1.44).

**Table 4:** Variance analysis (ANOVA) for PA-related knowledge, attitude, and behavior

		Group	Time	Group x Time
PA-related knowledge	*df1,2*	1, 142	1, 142	1, 142
	*F*	30.18	208.71	229.52
	*p*	**.000**	**<.001**	**<.001**
	*η*_p_^2^	.175	.595	.618
PA-related intention	*df1,2*	1, 142	1, 142	1, 142
	*F*	10.78	12.82	18.67
	*p*	**.001**	**<.001**	**<.001**
	*η*_p_^2^	.071	.083	.116
PA-related self-efficacy	*df1,2*	1, 142	1, 142	1, 142
	*F*	2.19	9.68	16.81
	*p*	.141	**.002**	**<.001**
	*η*_p_^2^	.015	.064	.106
Vigorous physical activity (min./week)	*df1,2*	1, 118	1, 118	1, 118
	*F*	.01	.55	.09
	*p*	.933	.459	.769
	*η*_p_^2^	.000	.005	.001
Moderate physical activity (min./week)	*df1,2*	1, 105	1, 105	1, 105
	*F*	.10	1.85	3.21
	*p*	.752	.177	.076
	*η*_p_^2^	.001	.017	.030
Minutes walked (min./week)	*df1,2*	1, 123	1, 123	1, 123
	*F*	.65	73.65	40.16
	*p*	.423	**<.001**	**<.001**
	*η*_p_^2^	.005	.375	.246

*Notes*: *df* = degree of freedom, *F* = test value, *p* = significance, *η*_p_^2^ = partial eta-squared; significant *p*-values (after correction for mass significance according to Bonferroni–Holm) are in bold print

Similar outcomes could be found for PA related intentions. Here again an improvement in participants intention to be physically active in future could be seen for the IG from t_1_ (*M* = 3.29, *SD* = .642) to t_2_ (*M* = 3.63, *SD* = .523). By contrast, the values in the CG between t_1_ (*M* = 3.21 *SD* = .473) and t_2_ (*M* = 3.18, *SD* = .500) did not change significantly. The interaction (group x time) was significant with a medium effect size (*F*(1, 142) = 18.67, *p* < .008, *η*p^2^ = .116) (Table 4 & Additional file 2: Fig. S2).

Finally, study results demonstrate that the participants of the IG developed a stronger intention to continue to exercise even if faced with obstacles (PA related self-efficacy) from t_1_ (*M* = 2.65, *SD* = .731) to t_2_ (*M* = 3.02, *SD* = .664). The outcomes of the CG, on the other hand, do not show any changes between t_1_ (*M* = 2.70, *SD* = .806) and t_2_ (*M* = 2.65, *SD* = .657). Here as well a significant interaction effect with a medium effect size could be determined (*F*(1, 142) = 16.81, *p* < .008, *η*p^2^ = .106) (Table 4 & Additional file 3: Fig. S3).

Regarding the intermediate behavioral outcomes, an interaction effect for low levels of PA (walking activities) could be determined. A significant improvement in the *time spent walking per week* (min/week) could be found in the IG from t_1_ (*M* = 401.55, *SD* = 208.03) to t_2_ (*M* = 526.48, *SD* = 240.56), while for the CG no change could be detected. The interaction effect was significant with a large effect size (*F*(1, 123) = 40.161, *p* < .008, *η*_p_^2^ = .246) (Table 4 & Additional file 4: Fig. S4). The mean increase of 125 min spent walking per week corresponds to an increase in low PA of about 30% in the IG (compared to 4.5% in the CG).

By contrast, no interaction or main effects could be found for *moderate and vigorous PA*, even though in the descriptive statistics there was an increase of 20 min/week for moderate PA in the IG over the intervention period (CG = 2.76).

## Discussion

The purpose of the present study was to evaluate the effectiveness of a tracking-based online intervention on the promotion of PA in worksite settings. In contrast to other interventions that emphasize vigorous or moderate PA, Healingo Fit focuses on low intensity forms of PA such as walking activities. The reason for this focus are data showing that worldwide less than one third of the adult population are physically active with high intensities on three or more days per week [8]. Promoting more vigorous forms of PA could thus be perceived as a too high barrier for many people.

Study results show that participation in the Healingo Fit intervention lead to a substantial increase in walking activities of about 30% in comparison to baseline. This percentage increase corresponds to the findings of Bravata et al.’s review [12] and the results of a further study of a worksite intervention in New Zealand, where the use of a pedometer in conjunction with the delivery of educational content over a period of 12 weeks lead to an increase in steps of 59% for the intervention group [27]. Considering the increasing evidence on the positive effects of low-intensity PA, these results are promising. Meta-analyses show that alongside a reduction of cardio-vascular risk factors (e.g. blood pressure, BMI, and body fat) there is a linear causal relationship between the extent and the intensity of walking and overall mortality [28, 29].

In addition to the behavioral effects, the evaluation also revealed positive effects on PA related knowledge, self-efficacy, and intentions. These effects are critically important and are the subject of numerous theoretical models. For example, the theory of planned behavior assumes that the implementation of a health behavior (TPB) is preceded by the formation of an intention, which in turn is predicted by intra- and interpersonal factors [18]. Empirical research has revealed that TPB-based interventions are effective in changing behavior and antecedent variables [30] and that medium-to-large changes in intention leads to small-to-medium changes in behavior [31]. TPB as well as other models of health behavior have guided the development of the intervention and a number of behavioral change methods derived from these theoretical models were used in Healingo Fit. These include e.g. methods of (1) information transfer (quizzes), (2) self-monitoring (e.g. visualized progress), (3) goal-setting and action-planning (health goals), and (4) persuasion (e.g. motivational messages in daily mails). In their recent Meta-Analysis, Steinmetz et al. [30] could show that persuasion had the greatest effect on antecedent variables such as intention which supports the use of those behavior change methods.

However, previous research has also argued for a discrepancy between the intention and health behavior (intention-behavior gap) and the need for constructs that mediate between these two. Across different studies, evidence supports the mediating role of self-efficacy and action-planning [32, 33]. The importance of self-efficacy as a determinant of PA is also highlighted in numerous studies [34], which is why Healingo Fit incorporates different forms of feedback and the visualization of participant progress (e.g. number of steps, progress reports, performance awards). However, with regard to action-planning Healingo Fit is limited to goal setting and provide only little support (e.g. tips by other participants) on implementation planning. Thus, the implementation of additional planning tools could be an useful extension which might increase intervention effects.

Finally, the features used in Healingo Fit are based on different forms of gamification (i.e. the application of classical game mechanisms to non-gaming contexts). Even though there are numerous successful implementations of gaming approaches in health promotion and disease prevention internationally, these are under-developed in German-speaking countries [15]. There are now different taxonomies of gamification strategies that are related to health behavior changes [35]. Many of these strategies (e.g. social connectivity in the form of support, and competition or reward mechanisms, especially awarding points) were integrated into Healingo Fit and used as motivational cues to support PA behavior. Initial evidence supporting this approach could be found in a current Dutch study on the effects of tracking devices [36]. Findings show that aspects of gamification, in comparison to other elements (e.g. comprehensibility or attractiveness), show the greatest explanatory power in predicting health-related experience of competency. Moreover, in their Meta-Analysis, Steinmetz et al. [30] provide preliminary evidence, that motivational behavior change methods were most successful for intention and direct behavior and hence could be regarded as a further mediator between intention and behavior.

A number of limitations should be considered when interpreting the results. Due to organizational reasons (time constraints of the cooperating partner) only two measurement points could be realized, and as a result findings about the intervention’s effectiveness are restricted to the point in time immediately after the intervention. Whether the effects sustain over longer time periods is thus still unknown, and further research is needed. In this context it should be noted that it cannot be ruled out that simply wearing the pedometer could lead to positive effects [37]. Unfortunately a third group condition (being equipped with a pedometer but not having access to the online intervention Healingo Fit) could not be implemented due to operational reasons and should be included in future studies. However, in most studies showing that pedometers are promising in increasing physical activity these devices are part of an intervention or a health-promoting program. In an evaluation of a study-led PA intervention including pedometers and behavior strategies for the IG and pedometers only for the CG, Raedeke et al. [38] conclude that “simply wearing a pedometer and keeping a step-log were not sufficient to promote activity”. Moreover, it must be taken into account that after expressing their interest in the intervention and taking part in the following random group allocation, 24 participants from the IG and 32 from the CG already dropped out before the intervention began. However, a comparison of the study participants in terms of socio-demographic variables revealed no systematic differences, which means that attrition bias can be excluded. Finally, all data on PA in this study are based on self-evaluation (IPAQ-SF), which could be influenced by memory bias or social desirability. Although comparison between the subjective reports and the objective recording of PA, using for example tracking devices, shows correlations in the expected direction [13], on average self-reporting indicates higher levels of PA [39]. To control for subjective overestimation, we recommend future research use both subjective and objective measures [39].

## Conclusion

In summary, this evaluation of Healingo Fit shows that a worksite intervention combining the use of pedometers with gaming elements in a theoretically based online intervention system can create positive effects in terms of PA-related knowledge, intention, self-efficacy, and PA behavior. Due to its online format Healingo Fit offers the potential for reaching large numbers of people and achieving population effects. The RCT design of this study is a considerable methodological strength and thus adds to the evidence supporting worksite interventions to promote PA. However, future research should (1) examine long term intervention effects and (2) use subjective and objective measure of PA.

## Additional files

Additional file 1: Figure S1. Pre- and post-intervention differences for PA related knowledge. Description: *M* = mean, scale range: 1 to 4, solid line represents the intervention group and dotted line the control group, interaction for group x time, *F*(1, 142) = 229.52, *p* < .008, partial eta squared of *η*_p_^2^ = .618 indicates a large effect (DOCX 23 kb)

Additional file 2: Figure S2. Pre- and post-intervention differences for PA related intention. Description: *M* = mean, scale range: 1 to 4, solid line represents the intervention group and dotted line the control group, interaction for group x time, *F*(1, 142) = 18.67, *p* < .008, partial eta squared of *η*_p_^2^ = .116 indicates a large effect (DOCX 24 kb)

Additional file 3: Figure S3. Pre- and post-intervention differences for PA related self-efficacy. Description: *M* = mean, scale range: 1 to 4, solid line represents the intervention group and dotted line the control group, interaction for group x time, *F*(1, 142) = 16.81, *p* < .008, partial eta squared of *η*_p_^2^ = .106 indicates a medium effect (DOCX 24 kb)

Additional file 4: Figure S4. Pre- and post-intervention differences for total minutes walked (min/week) Description: *M* = mean, solid line represents the intervention group and dotted line the control group, interaction for group x time, *F*(1, 123) = 40.161, *p* < .008, partial eta squared of *η*_p_^2^ = .246 indicates a large effect (DOCX 24 kb)

## Abbreviations

ANOVA: analysis of variance
CG: control group
HAPA: Health Action Process Approach
Healingo: Health Integrated Gaming Online
IG: intervention group
PA: physical activity
RCT: Randomized Controlled Trial
TPB: Theory of Planned Behavior
WHO: World Health Organization
