# Supplementary material for: Promoting physical activity in worksite settings: results of a German pilot study of the online intervention Healingo fit
Source: BMC Public Health. 2017 Sep 8;17:696. doi: 10.1186/s12889-017-4697-6 (PMC5591504; doi:10.1186/s12889-017-4697-6)
Supplement: Supplementary file 4 [file 12889_2017_4697_MOESM4_ESM.docx]

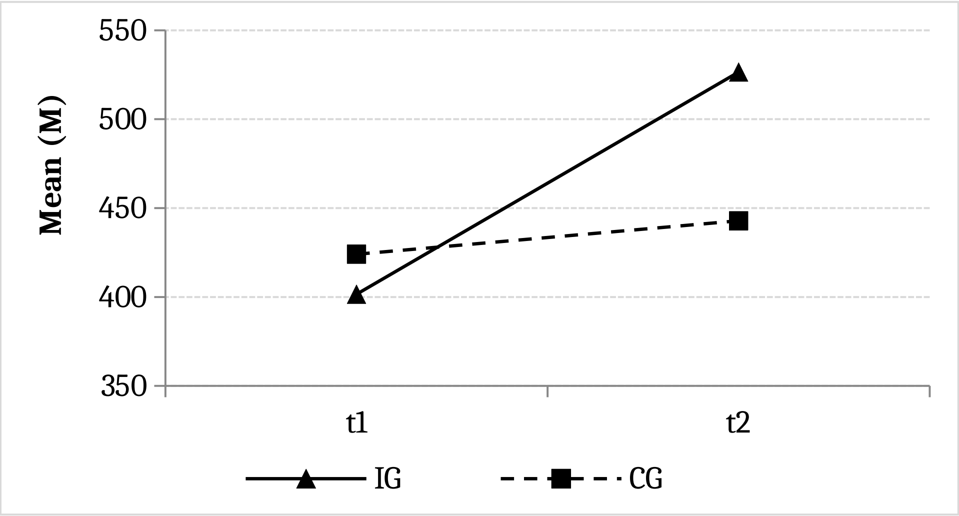
550
500
450
400
350
Mean (M)
t1
t2
IG
CG
